# Opportunistic and On-Demand Network Coding-Based Solutions for LPWAN Forwarding

**DOI:** 10.3390/s20205792

**Published:** 2020-10-13

**Authors:** Dion Tanjung, Seunggyu Byeon, Junhwan Huh, Dong Hyun Kim, Jong Deok Kim

**Affiliations:** 1 Department of Electrical and Computer Engineering, Pusan National University, Busan 46241, Korea; tanjung.dn@pusan.ac.kr (D.T.); ijhhuh10@pusan.ac.kr (J.H.); dhkim1106@pusan.ac.kr (D.H.K.); 2 Division of Computer Software Engineering, Silla University, Busan 46958, Korea; sg0919@silla.ac.kr

**Keywords:** LPWAN, relay, on-demand, unnecessary forwarding, opportunistic forwarding, network coding, compressed header overhead

## Abstract

The single-hop star-of-stars topology in low-power and wide-area networks (LPWAN) exhibits reliability and substandard coverage issues, especially in urban areas where line-of-sight (LoS) communication is difficult to achieve. Moreover, LPWAN trade-off the data rate to achieve longer coverage, preventing other end-devices from using the time resource. Locating other gateways is uneconomical as it requires infrastructure, such as the internet and a power connection. In this study, we propose a forwarding scheme with a relay to increase LPWAN coverage and reliability while not degrading the network’s capacity. A relay tends to incur unnecessary forwarding that degrades the network capacity unless proper countermeasure is prepared. Our works, namely opportunistic and on-demand network coding (OODC), minimize unnecessary forwarding and make good use of multiple-receiving relays. Network coding is also applied in the relay for better transmission efficiency and reliability simultaneously. Because network coding occurs header overhead, we perform a header compression technique to counter it. According to our simulation result, our method shows better reliability than fixed path forwarding. In an adaptive data rate settings, the OODC achieves a 92% packet delivery ratio (PDR), whereas a fixed-path forwarding only achieves an 84% PDR.

## 1. Introduction

Low-power and wide-area networks (LPWAN) technology has gained significant attention from the standpoint of IoT services as it facilitates long-range communication with low-power consumption. This technology works on the sub-GHz band, allowing wider coverage up to 20 km in the line-of-sight (LoS). Typically, the data rate is limited to 0.3 kbit/s to 50 kbit/s [1]. However, it is sufficient for internet-of-things (IoT) applications, such as smart city or telemetry services that send small packets over wide areas.

Several LPWAN technologies are available in the market, based on licensed bands (LTE-M and NB-IoT ) and unlicensed bands (Sigfox and LoRa). Among them, LoRa [2] or long-range radio, using a CSS (Chirp Spread Spectrum) modulation technique, is a popular LPWAN radio technology that has been adopted worldwide. LoRaWAN [3] is a Medium Access Control (MAC) layer specification used to build LPWAN networks based on LoRa radio. LoRaWAN is gaining popularity because it is an open standard that allows researchers and developers to improve and modify the specifications according to their needs.

LoRaWAN adopts single-hop star-of-stars network topology where the end-devices transmit its packet directly to one or many gateways via LoRa modulation. The gateway acts as a bridge forward the received packet to the network server using a standard IP connection. Although simple and robust, it trade-off the data transmission rate to achieve longer coverage. There are 6 available spreading factor (SF) (7–12) and 3 Bandwidth (BW) selection (125 kHz, 250 kHz, and 500 kHz) [3]. As the SF is increased and the BW is decreased, greater coverage can be achieved. However, the use of a slower data transmission rate in LoRaWAN with an Aloha based protocol increases the collision probability. Field-testing conducted by Augustin et al. [4] shows that SF 12 is required to achieve a 90% packet delivery ratio (PDR) for coverage below 3 km. However, this low data rate settings can only support 120 end-devices [5].

Stationing more gateways near the end-device may solve reliability and scalability issues in LoRaWAN. However, a gateway requires internet access, which increases cost and not suitable for a location where internet infrastructure is extremely limited (e.g., basements). An alternative solution is to employ a forwarding mechanism by placing a relay between the end-device and the gateway.

In this study, we investigated the benefit of stationing a relay between the end-devices and a gateway. The use of relay extends the coverage and, in addition, increases the scalability via utilization of a faster data transmission rate. However, random placement of the end-devices in a real-world case makes it hard to put the relay at the right location. Each end-device facing different interference, and some of them could transmit packet directly to the gateway using the best data rate. Although a packet duplication caused by unnecessary forwarding can increase reliability [6,7], it also reduces the availability of network resources that prevent transmission from the end-devices. For a massive IoT, this unnecessary forwarding can be a severe problem that decreases the overall reliability.

**Figure 1:**
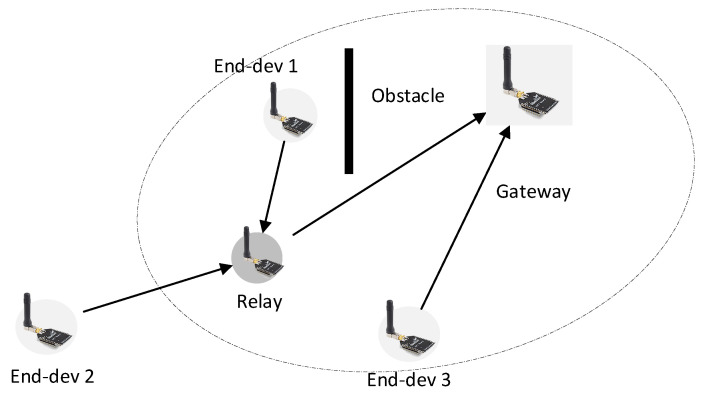
Ideal use of a relay. Not all end-devices require relays to preserve reliability. Relay is only useful for extending coverage and forwarding packet from a blocked end-device.

Motivated by the side effect of unnecessary forwarding, we design a relay forwarding mechanism to forward a packet from the end-devices that cannot communicate directly to the gateway, as shown in Figure 1. We also consider that wireless communication is unpredictable and showing poor dynamic characteristics owing to dynamic propagation [8]. Sometimes, the end-device can transmit directly to the gateway. However, when a forwarder is needed, the selected relay can fail to receive the packet. When several relays are present, it is beneficial to utilize multiple-receiving of one or more relays that overheard the packet. Instead of asking retransmission from the end-devices that are unnecessary, any relay that receives the packet can forward the packet. We cannot rely on retransmission as the strict regulation of 1% duty-cycle constraint makes acknowledgment-based ineffective because the gateway has to send ack at the specified time-slot.

The majority of previous LPWAN forwarding studies are based on fixed-path forwarding that immediately forwards the received packets via a predetermined path [9,10,11,12,13]. The other studies adopt flooding transmission that allows synchronized packet collision [14,15]. However, both approaches only consider improving high reliability and low latency. They do not consider the trade-off between network reliability and capacity issues caused by unnecessary forwarding. This paper presents opportunistic and on-demand network coding (OODC) forwarding to increase the LPWAN coverage with improved reliability and transmission efficiency. As the header overhead of network coding may exceed the size limitation of the LPWAN packet, we use a header compression technique to counter the increase. The contributions of our work with respect to LPWAN forwarding are as follows:

- Adoption of opportunistic forwarding that considers transmission over a shared wireless environment. Without predetermining a forwarding path, we allow any relays to forward the packet. Hence, we can increase transmission reliability and network throughput.
- Adoption of on-demand/feedback message based forwarding in the relays instead of proactive forwarding to prevent unnecessary duplicate forwarding.
- Adoption of a random linear network coding (RLNC) to tackle the bottleneck problem in opportunistic forwarding. Because of the LPWAN packet size limitation, we employ header compression to reduce RLNC header overhead.
- Through simulation, we show that a relay increases the network coverage and improves the network reliability with better efficiency by utilizing a multiple fast data rate instead of a single slow data rate.

The paper goes on to discuss related works in Section 2, motivation for LPWAN forwarding concept in Section 3, study on LoRa in Section 4, design and forwarding operations in Section 5, and implementation and evaluation in Section 6, followed by conclusions and future directions of work.

## 2. Related Works

### 2.1. Forwarding Architecture

**Figure 2:**
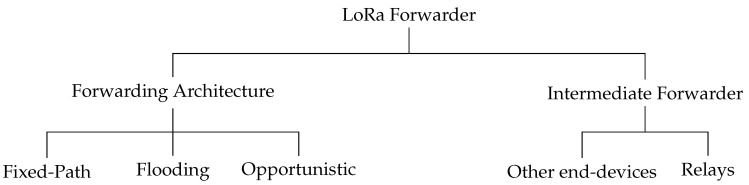
LoRa forwarder anatomy.

There are three kinds of multi-hop approaches for LPWAN forwarding: fixed path, flooding, and opportunistic, as shown in Figure 2. Fixed-path forwarding is a topology based routing, according to which each node exchanges information to maintain a routing table. We group the routing discovery into proactive-based (Destination-Sequenced Distance-Vector (DSDV) and Optimized Link State Routing (OLSR)), reactive-based (Dynamic Source Routing (DSR) and Ad-hoc On-demand Distance Vector (AODV)), and Hybrid-based (Hybrid Wireless Mesh Protocol (HWMP)) [16,17,18]. Fixed-path forwarding determines the best path using routing metrics, such as Expected Transmission Count (ETX) and Expected Transmission Time (ETT) [19,20,21,22]. The LPWAN multi-hop solutions in [9,10,11,13] are based on routing protocol for low-power and lossy networks (RPL) routing. The authors in [12] adopt HWMP hybrid forwarding to reduce high control overhead in the proactive-based and high latency in the reactive-based routing discovery mechanism. However, this fixed-path based approach shows poor performance in an unstable wireless environment, especially when the next destination cannot receive the transmitted packet [8].

The combination of flooding architecture and concurrent transmission (CT) is presented in [23]. The flooding originates from a transmission broadcast by an initiator; subsequently, any receiver will rebroadcast the packet immediately as the next wave, which leads to a broadcast storm problem [24]. In LPWAN multi-hop, this approach is adopted by LoRaBlink [14] and CT flooding [15]. Unlike LBT (Listen Before Talk) or CSMA/CA (Carrier-sense multiple access with collision avoidance) [25] that avoids collisions, CT synchronizes the duplicate packet to recover the packet. Although CT exhibits excellent performance in energy consumption, reliability, and latency, flooding transmission reduces network capacity.

ExOR [8] is the first conception of opportunistic routing that exploits multiple-receiving relays to forward an overheard packet based on a strict timing schedule to prevent collisions. This approach is proved to increase network reliability and throughput more efficiently. A Simple Opportunistic Adaptive Routing (SOAR) [26] improves Exor by reducing the diverging path and removing strict scheduling. In SOAR, each packet brings a list of relay candidates calculated from the ETX metric. The relay candidates coordinate and exchange transmission information state to prevent duplication. A Cooperative Power and Energy-efficient routing (COPE) [27] introduces network coding to prevents strict regulation. Each relay encodes multiple packets together, and then intelligently transmit the coded packets and reuse the duplicate packets for facilitating packet recovery. The authors in [7] propose a cooperative relaying for the LoRa network by exploiting the benefit of packet redundancy. Instead of performing a routing mechanism, the relay combines the overheard packet and then forwards it to the gateway following the duty cycle limitation. Although it achieves high-reliability performance, this approach is only good to combine a small packet of sensor data in a short distance. As it uses the maximum size of LoRa payload for packet relaying, these works consume a high amount of time resources in a higher SF.

### 2.2. Intermediate Forwarder

We can use other end-devices or place a relay between the end-device and the gateway for the intermediate forwarder. Hare [9] topology is similar to a hierarchical tree that uses end-devices close to the gateway as the intermediate node. This topology requires time synchronization, such as time division multiple access, to synchronize Transmission Tx and Reception Rx between the parent and its children. Similarly, the authors in [13,14,28] also propose a solution that requires a parent to forward a packet and time synchronization to avoid a collision. However, both approaches will pressure the parents because they have to listen and forward several packets from their children.

The authors of [10,11,12] use a relay to forward a packet from the end-device to the gateway. Although adding a relay incur a financial cost, this does not put pressure on the other end-device, and we can exclude the end-device from the routing discovery. Besides, this relay is more economical than putting an extra gateway as it is cheaper and does not require internet access.

### 2.3. Proposed Solutions

This work is an improvement of our previous study [29] that we propose different solutions by employing on-demand, opportunistic forwarding, and network coding approach. Random Linear Network Coding (RLNC) is a network coding approach that encodes a linear combination of multiple packets with a coefficient selected from the Galois Field (GF). This packet can be decoded if the receiver receives sufficient linear independent combinations. However, the coefficient needs to be included in the packet header, resulting in header overhead that can exceed the maximum packet size allowed in LPWAN. Many researchers present a compression strategy by limiting the number of combined packets to *m* [30,31]. The authors of [30] improve the compression strategy by using a pair of a small set of the allowed coefficient from the GF.

## 3. Challenges and Motivation

### 3.1. Preventing Unnecessary Forwarding

One of the side effects of the forwarder mechanism is packet duplication caused by unnecessary forwarding. Unnecessary forwarding reduces the network capacity, which prevents packet transmission from an end-device, thereby leading to performance degradation. As LPWAN already facilitates broader coverage, the forwarder mechanism is only useful under certain conditions (see Figure 1).

An LPWAN forwarder should consider any further forwarding as unnecessary once the gateway has already received the packet. To prevent unnecessary forwarding, we present an on-demand mechanism via feedback messages similar to SACK (selective acknowledgment), containing details about all currently received packets. Thereby, the relay only forwards the missing packets after receiving and checking the packet’s state from the network server.

### 3.2. Reliability in Uncertain Wireless Conditions

**Figure 3:**
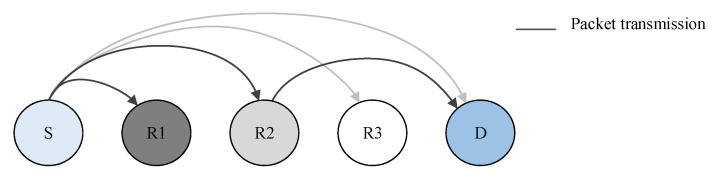
Uncertainty of wireless transmission.

As the wireless link is unstable, Figure 3 shows the possible transmission and reception between an end-device, several relays, and a gateway in LPWAN cases. Let us suppose that R1 and R2 receive the message, while R3 fails owing to the propagation problem. If fixed-path routing assumes that R3 is the best forwarder, it is not delivered to the destination. Even if the gateway can acknowledge the loss packet and request retransmission, commonly, the request message has to be forwarded via R3 during the end-device’s receiving window period. This kind of process is inefficient.

We employ opportunistic forwarding that allows any relay to forward an overheard packet via priority mechanisms to prevent packet duplication between the relays. According to Figure 3, R2 can forward the packet after noticing that R3 of higher priority, fails to deliver the packet. Therefore, we can achieve better reliability and throughput than that of fixed-path forwarding.

### 3.3. Bottle Neck in Opportunistic Forwarding

Opportunistic forwarding only improves transmission reliability between the sender and intermediate forwarder. However, packet forwarding from the relay to the gateway may fail, creating a long queue in the relay that causes a bottleneck problem, especially if the relay requires to retransmit the packets loss in the next feedback message. Therefore, we employ a network coding approach in the relay that can increase network throughput and reliability via encoding and decoding multiple packets.

### 3.4. Header Overhead of RLNC in LPWAN

According to the studies on LoRaWAN [4,32], the available LoRaWAN payload size varies between 51 and 250 bytes with the header of 13 to 28 bytes depending on the data rate settings. Because the header overhead of RLNC is $n\;log_{2}\;q$ bits, where n is the number of packets and *q* is bit size in $GF(2^{q})$, we cannot implement RLNC directly in a massive IoT network. For example, a relay using $GF(2^{4})$ to encode 100 packets of 30 bytes in payload size will have a coded packet of 50 bytes header that cannot be supported in some data rate settings considering the packet size regulation. Therefore, we adopt overhead header compression to compensate for packet size limitation in typical LPWAN.

## 4. LoRa Study

### LoRa Link Behavior

LoRa transmission is successful if the link budget or received signal power $P_{rx}$ greater than the receiver sensitivity $S_{rx}$. The Sensitivity $S_{rx}$ depends on the LoRa SF and Bandwidth:

(1) $$S_{rx}=-174+10log_{10}BW+NF+SNR$$

where −174 is thermal noise in a 1-Hz bandwidth, BW is the receiver bandwidth, NF is a fixed value for receiver noise figure, and SNR is the ratio of signal power to the noise level as determined according to the selected spreading factor. The higher is the SF, the more sensitive is the receiver; thus, the receiver could receive a packet from a greater distance.

Total gains and losses determine the link budget during the transmission path can be expressed as follows:

(2) $$P_{rx}=P_{tx}+GL-L_{pl}$$

where $P_{tx}$ is the sender transmission power, GL is the combination of all general gains and losses, and $L_{pl}$ is path loss in the wireless medium.

To calculate the path loss, we use a log-distance path loss model [33]:

(3) $$L_{pl}\left(d\right)=\bar{L_{pl}}\left(d_{0}\right)+10\gamma Log_{10}\left(\frac{d}{d_{0}}\right)+X\sigma$$

where $L_{pl}\left(d0\right)$ is the mean path loss at reference distance $d_{0}$, *d* is distance, γ is the path loss exponent, and $X\sigma ∼(N,\sigma^{2})$ is the Gaussian distribution with a zero mean. To make our simulation more realistic, we provide the PDR acceptance model by observation:

(4) $$PDR\left(\hat{P_{rx}}\right)=Pr\left(\hat{P_{rx}}>S_{rx}\right)=P_{r}\left(z>\frac{S_{rx}-\hat{\mu }}{\hat{\sigma }}\right)$$

where *z* is Gaussian distribution, $\hat{\mu }$ is the mean of the observed $P_{rx}$, $S_{rx}$ is the receiver sensitivity, and $\hat{\sigma }$ is the observed standard deviation.

The empirical measurement of LoRa transmission has been discussed in Reference [34], with γ = 2.32, $LPL(d_{0})$ = 128.95, and $X_{\sigma }$ = 7.8 dB. We also considered the measured receiver sensitivity on SX1272 Dataset [35].

## 5. Design

### 5.1. Feedback/On-Demand Forwarding

According to our mechanism, the relay starts to forward after receiving a feedback message from the network server. This feedback message contains a list of current packets received by the network server from the previous feedback. Each packet is identified by packet id and sequence number, allowing the LoRaWAN packet to bring a long list without exceeding the LoRaWAN packet size’s maximum capacity. This packet ID is generated by the network server during the join process, derived from a unique device ID given by the manufacturer.

To avoid collisions between feedback messages and forwarding messages or packet messages, we utilize separate channels. Any relay that receives a feedback message will do the filtering process:

- Packet Filtering: The relay compares the packet id and packet sequence of the received packets to the feedback message’s information. It selects only the missing packets from the feedback message and then forwards them to the gateway. However, the selected packets will not be removed as it can be used for the next feedback process if the packet is still lost. The packet is removed from the relay if it is included in the feedback message.
- Time Filtering: There are cases wherein the gap between the feedback message transmission time in the network server and its reception time at relays may lead to unnecessary forwarding. Suppose that the network server already generated a feedback message without including a packet that had just arrived. If the relay receives this feedback message, it unnecessary forward the late packet. Therefore, a packet that is received during feedback message transmission will be forwarded in the next feedback message.

### 5.2. Opportunistic Forwarding

The main aim of using opportunistic forwarding is to exploit multi-reception benefits for increasing network reliability and network throughput. However, the forwarders need to coordinate each other to prevent duplicate packet forwarding. The coordination mechanism involves listening transmissions from other relays and prioritizing relay:

- Opportunistic listening: A relay always listens to transmissions by other relays. If the higher prioritized relays do not forward a packet, the relay can forward it to the gateway.
- Priority forwarding: The priority mechanism is a critical aspect for preventing redundancy and collision. As per our mechanism, relay priority is based on its Received Signal Strength Indicator (RSSI). The network server is responsible for managing this priority. The network server collects this RSSI and saves it to the correct relay id in the priority list. Because of different RSSI measurements in every transmission, the network server always takes the RSSI average before sorting the priority list. The gateway sends this priority list before sending the feedback message because the packet information list already occupies feedback capacity. After receiving the feedback message, the relays forward the received packets as per these priority rules.

**Figure 4:**
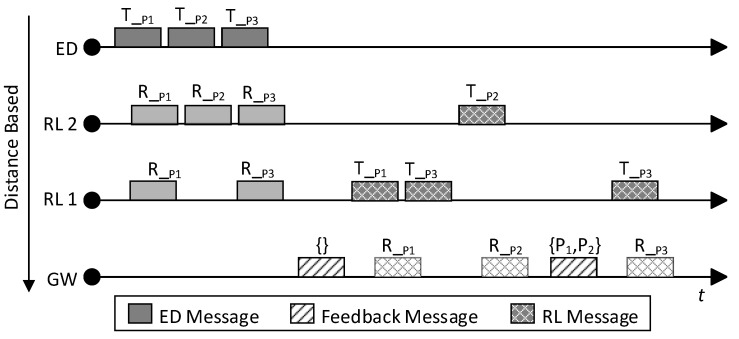
Example to illustrate transmission priority and feedback message.

Figure 4 illustrates the transmission of feedback messages and forwarding messages. The Y-axis represents the distance between each device and the gateway. Consequently, relay 1 has a higher priority than relay 2. First, an end-device transmits (T) 3 packets via LoRa communication. Relay 2 receives (R) all packets successfully, while relay 1 only receives packets P1 and P2. Because the gateway does not receive any packets, it sends an empty feedback message. After relay 1 receives the feedback, it begins to transmit packets P1 and P3 while relay 2 continues to wait and listen. Relay 2 suppresses forwarding of P1 and P3 as it overhears the forwarding of them by relay 1, but it forwards P2 as there is no forwarding of P2 by other relays. The next feedback message contains P1 and P2 without P3 because it was lost during forwarding transmission. Lastly, only relay 1 forwards packet P3.

### 5.3. Network Coding

Random Linear Network Coding (RLNC) is one of the network coding strategies that combines packets with coefficients selected from a Galois field (GF) to create random linear combinations. The sender initially encodes multiple packets of $P_{n}$ to generate *k* random linear combinations of $P^{\prime }$ by:

(5) $$P_{k}^{\prime }=\sum_{n=1}^{k}c_{n}*P_{n}$$

where $c_{n}$ is the selected coefficient for each packet. The sender transmits this linear combination along with its selected coefficients in the header. Thus, it increases the header overhead. The sender transmits these random linear combinations until the receiver obtains linearly independent combinations for successful decoding. The receiver decodes the combination via Gauss-Jordan elimination method [36]:

(6) $$\left|\begin{array}{c} P_{1}\\ :\\ P_{k} \end{array}\right|={\left|\begin{array}{ccc} c_{11} & .. & c_{1k}\\ : & .. & :\\ c_{k1} & .. & c_{kk} \end{array}\right|}^{-1}\left|\begin{array}{c} P_{1}^{\prime }\\ :\\ p_{k}^{\prime } \end{array}\right|$$

#### 5.3.1. Header Overhead Compression

The header overhead in RLNC depends on the number of coefficients included in the packet combination. Therefore, we limit the number of possible packet combinations as *m* and use a subset of primitive pair elements from the GF as *Q* [30].

Initially, we set *m* and *Q* as shared variables between the relay and the network server. Then, for every *n* packets, relays generate a k∗n (k>n) sparse matrix of linearly independent combinations. The symbol *k* is an additional number of combinations, calculated via PDR estimation between the relay and gateway.

For example, let us consider that there are six packets in the relays encoded by m=3 and Q=[4,14] from GF(16) that represents coefficient Q=[γ,α]. The generated matrix is:

$$E_{k}{*}_{6}=\left|\begin{array}{cccccc} 0 & 0 & 4 & 0 & 4 & 14\\ 14 & 0 & 4 & 14 & 0 & 0\\ . & . & . & . & . & .\\ 0 & 4 & 4 & 0 & 4 & 0 \end{array}\right|$$

The non-zero values represent the combination of *m* packets with the coefficient selected from *Q*. Before transmission, the relay applies Compressed Sparse Row (CSR) format to identify and include only the non-zero element of each row vector. The header $H=(I_{1}||J_{1}||S_{1}||\ldots ||I_{i}||J_{i}||S_{i})$ where Iμ is the coefficient index in Q[0,1], Jμ is the packet id, and Sμ is the packet sequence number.

For the purpose of illustration, let us consider that the header of the first row vector is to be formatted as per the previous example; if θ is assumed to be the packet sequence number, the compressed header will be h=[0||010||θ||0||100||θ||1||101||θ]. Hence, the relay transmits both the header *H* and the linear combination $P^{\prime }$ to the gateway.

If we assume the packet id represents the end-device index position in a shared list between the relay and the network server, packet id size is $log_{2}\;n$ bits. In combination with the 16 bits of the packet sequence number [3] and 2 bits of *Q* size, the header overhead is $m(17+Log_{2}N)$ bits.

#### 5.3.2. Transmission Strategy

Multi-reception enables each relay to receive a duplicate packet from the end-devices. In order to reduce network traffic, we utilize an overhearing strategy.

- Identical packets: Here, all packets in two relays are duplicated. Therefore, only relay with higher priority forward the combined packets.
- Intersection packets: Here, not all packets in the relays are duplicated. Hence, the relay with the lower priority should only transmit the linear combinations that constitute a missing packet from another relay.
- Without intersection: Here, all the packets in relays are different. Therefore, both relays may transmit all of the generated combinations.

#### 5.3.3. Decoding Process

The network server generates a list of submatrix that are used to store the incoming coded packet $P_{c}$ from the gateway. As shown in Algorithm 1, the network server checks partial packets, a list of the end-devices packets used to form the coded packet. If any partial packets of $P_{c}$ are a subset of that in a submatrix that already created, we append $P_{c}$ to it. Otherwise, we create a new submatrix containing $P_{c}$.
**Algorithm 1** Decoding procedure**Phase 1—Slicing procedure**1:**procedure**submatrix = SLICING($P_{c}$,submatrix)2:    **if** submatrix is undefined or $P_{c}$ is independent to submatrix **then**3:        New submatrix← new List($P_{c}$)4:    **else**5:        submatrix.append($P_{c}$)6:    **end if**7:**end procedure****Phase 2—Decoding procedure**8:**procedure**packets = Decoding(submatrix)9:    sparsematrix← decompress header of each $P_{c}$ in submatrix10:    **if** sparsematrix is full rank **then**11:        Decode sparsematrix by Equation (6)12:        Remove submatrix13:    **else**14:        Request additional $P_{c}$15:    **end if**16:**end procedure**

The decoding process is started when there is no incoming coded packet during a timeout. For each submatrix, we create a sparsematrix by decompressing header of each coded packets in the submatrix. If the sparsematrix is full rank, the network server decodes it using Equation (6). Otherwise, the network server requests additional coded packets that contain the partial packets from all relays that generate these coded packets.

**Figure 5:**
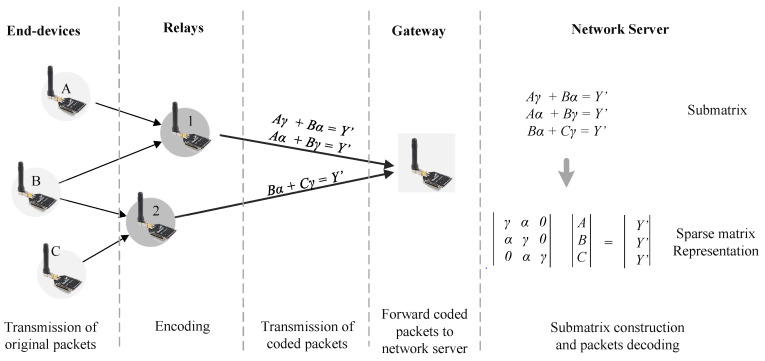
Network coding could recover linearly independent coded packets from different relays.

For example, the network server receive two headers of coded packets h=[1||001||θ||0||010||θ], h=[0||001||θ||1||010||θ] from relay 1 and one header h=[0||010||θ||1||011||θ] from relay 2 (see Figure 5). Originally, each sender only combines two packets, namely packets A and B in relay 1 and packets B and C in relay 2. During the slicing process, the network server merge these coded packets because they have similar partial packet B. Hence, the submatrix contains partial packets A, B, and C. After timeout, the network server starts decompressing the header to generate an $E_{3*3}$ sparse matrix:

$$E_{3*3}=\left|\begin{array}{ccc} 4 & 14 & 0\\ 14 & 4 & 0\\ 0 & 4 & 14 \end{array}\right|$$

Because the generated matrix is full rank or linearly independent, the network server decodes it as per Equation (6).

## 6. Implementation and Evaluation

### 6.1. Implementation Detail

We evaluated the proposed method performance using a simulator program that is built based on the LoRaSim [5]. This simulator mimics LoRa communication behavior, including its communication range and collision behavior through LoRa design [5,35,37].

For the evaluation, we used three data-rate scenarios: LoRaWAN slow data-rate (SDR), fast data-rate (FDR), and adaptive data-rate (ADR). Each scenario has different configurations of bandwidth (BW) and spreading factor (SF), as shown in Table 1. While both SDR and FDR apply static configurations, ADR scenario calculates the best SF to transmit the packets efficiently (i.e., utilize faster transmission). These different configurations affect not only the transmission range but also transmission time. According to communication range calculation, the transmission range in the SDR scenario is estimated to be up to approximately 8900 m, with a transmission time $T_{frame}$ of 1712.13 ms. Conversely, FDR may have a smaller coverage that is approximately 1100 m. However, the transmission time is only 14.14 ms.

The end-device packet is generated following a Poisson distribution with an average inter-packet time lambda = 16.7 m. In our simulations, the transmissions from the end-devices are not active during the whole simulation run-length time. End-devices are active only for the first *T* long interval after the start, leaving a short additional time for the relay to forward the remaining packet. During this *T*, the feedback message is transmitted periodically to the relay to start forwarding the missing packets. We performed 10 simulation for each evaluation and took the average to provide the results.

### 6.2. Evaluation Matrix

This evaluation aims to demonstrate the OOD (opportunistic and on-demand) and OODC (opportunistic and on-demand network coding) capabilities for increasing the LPWAN coverage and reliability while preserving the network capacity. We compare our work with FP (fixed-path) forwarding that represent the majority of relay forwarding protocol used by other related work. For each simulation, we increase the number of end-devices to evaluate scalability performance. We used PDR to evaluate performance with regard to extending LoRaWAN coverage and scalability. The PDR defines the ratio of successfully received packets in the network server to the transmitted packets from end-devices over a period. Conversely, the number of duplicated packets in the network server shows unnecessary forwarding from the mechanism. By Evaluating the effect of unnecessary forwarding to reliability performance, we can show the trade-off between network reliability and efficiency.

**Figure 6:**
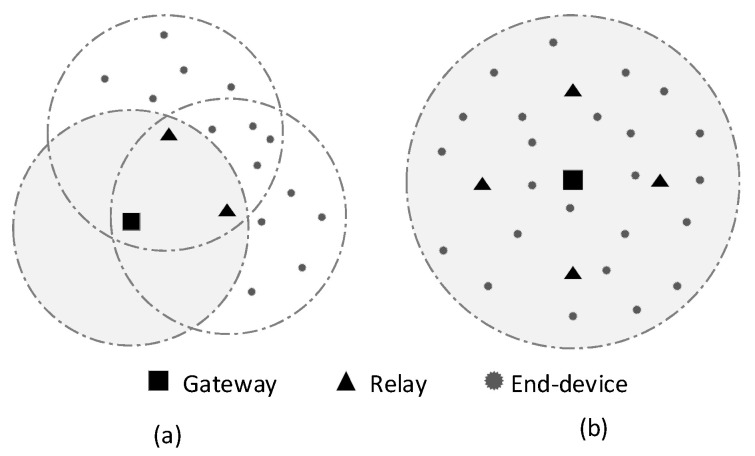
Two network topologies for the experiment. (**a**) Topology 1: The end devices are outside the gateway coverage. (**b**) Topology 2: The end devices are inside the gateway coverage.

For these purposes, we present two experiments with different network topologies. First, we focus on the performance of OOD, OODC, and FP in increasing LoRaWAN coverage via network topology 1 in Figure 6a. In this topology, there are two relays close to the border that can communicate with each other. The end-devices are placed outside gateway coverage according to the $P_{rx}$ estimation. Conversely, the second experiment is used to show the benefit of the relay to make efficient use of network resources via ADR mechanism. Thus, it can increase network scalability. The network topology 2 in Figure 6b consists of four relays and several end-devices inside the gateway coverage. The coverage is based on SDR settings to make the devices capable of choosing between SF 7 and SF 12 in ADR scenario. While the gateway coverage is approximately 8900 m, the relay distance to the gateway is approximately 4000 m.

### 6.3. Evaluation

#### 6.3.1. Studying LoRa Reliability

LoRaWAN regulation in South Korea allows choosing between 1% of duty-cycle and LBT approach in order to use the physical medium. This duty-cycle is good for saving the energy, suited for the end-devices that only wake up and transmit when it has a packet. Conversely, the relay is an always-on device that is connected to a good power source. For achieving good reliability, the LBT approach is preferable as it can avoid collision by sensing the medium.

In this part, we present a comparison between two different LoRa MAC protocols, pure-ALOHA and LBT-ALOHA. In the LBT-ALOHA, LBT performs channel activity detection (CAD) to detect any ongoing transmission in the medium before starting a transmission. If a preamble is detected during CAD, it performs exponential random back-off until the channel is free to avoid a collision. In this evaluation, we used the SDR scenario because it facilitates a longer transmission time suitable for showing collision problems. For the network topology, we put end-devices randomly in a single gateway coverage without a relay.

The traffic load is represented as $G=\lambda T_{frame}$, where λ=n/T is the input rate, *n* is the number of transmitted packets from the end-devices during simulation period *T*, and $T_{frame}$ is packet transmission time. There were approximately 760 packets transmitted from 100 end-devices and 8960 packets transmitted from 1200 end-devices in the simulation. The mean traffic load *G* is 0.1 and 2.1, respectively. In this case, a higher *G* represents a higher traffic load.

**Figure 7:**
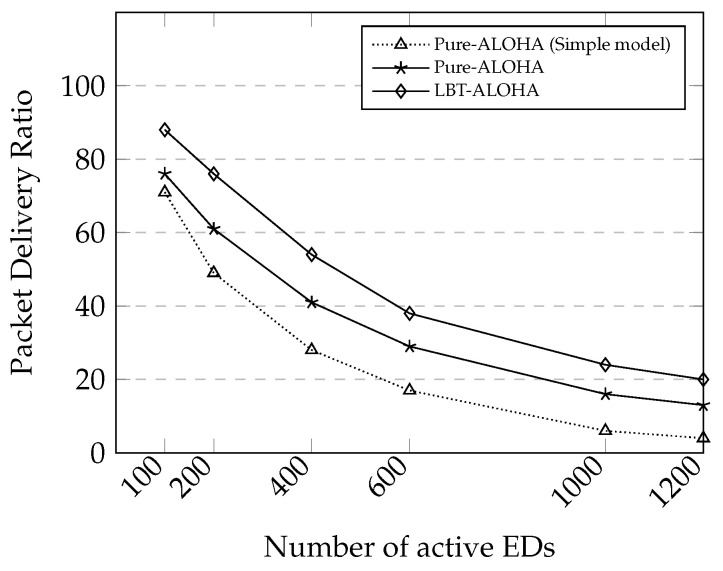
Packet delivery ratio (PDR) comparison between Pure-ALOHA without capture effect and Pure-ALOHA and LBT-ALOHA with capture effect. The capture effect and LBT approach contribute to in improving LoRa reliability.

It is well known that the capture effect, a phenomenon that a strong signal can survive in a collision by suppressing other weaker signals when overlapping at a receiver, can increase the overall packet delivery ratio (PDR) in LoRa communication [5,38]. Our simulation results also support it. As shown in Figure 7, the pure-ALOHA protocol with the capture effect has a higher PDR than that without the capture effect that is denoted as a simple model.

As the LBT-ALOHA can reduce the probability of collision through CAD, we can expect that the reliability of the LBT-ALOHA would be higher than that of the pure-ALOHA. As conforming to the expectation, our simulation results show that the PDR of LBT-ALOHA is higher than that of pure-ALOHA by approximately 12%. We applied the LBT-ALOHA with the capture effect in our simulations below.

#### 6.3.2. Increasing LoRaWAN Coverage

**Figure 8:**
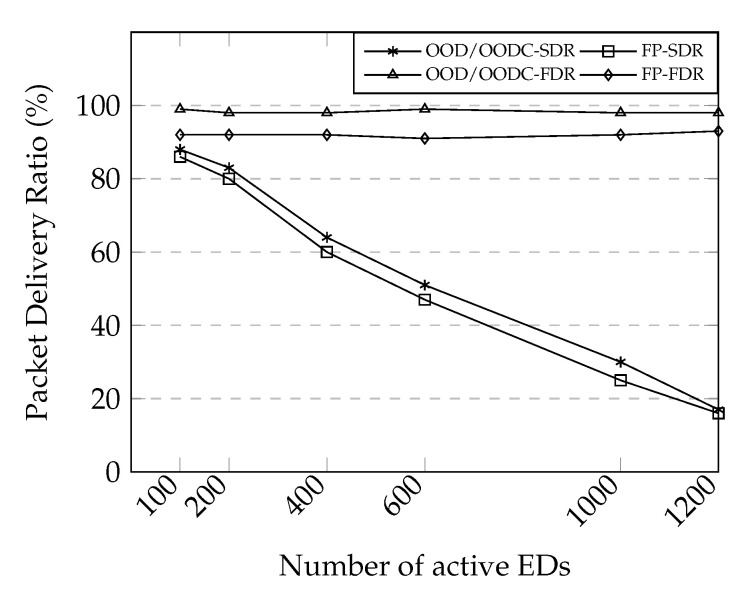
Performance evaluations on network topology 1. Reliability comparison between OOD, OODC, and FP forwarding on slow data rate and fast data rate scenarios. Here, multi-reception approach improves the reliability of OOD and OODC.

We evaluated our forwarding solutions’ reliability compared to that of fixed-path forwarding using the network topology in Figure 6a. Actually, the topology is not realistic as all end-devices are located out of the gateway’s coverage. However, it is a good topology to evaluate the extension of coverage through a relay. As shown in Figure 8, both OOD and OODC showed a similar result with better reliability compared to that of fixed-path forwarding in both SDR and FDR scenarios. This was mainly owing to the multi-reception benefits of opportunistic forwarding.

Under fast data rate settings, both OOD and OODC maintained performance above 90% of the PDR, even increasing the number of active devices up to 1200. Conversely, both solutions can serve only up to hundreds of end-devices under slow data rate settings to achieve similar reliability performance. This result shows that the difference in transmission times between the SDR and FDR settings affects the network capacity. As more end-devices are in the contention, the packet collision increases.

#### 6.3.3. Improving LoRaWAN Scalability

The previous evaluation shows that network resources are important in LPWAN. According to [5], the adaptive data rate (ADR) can increase LoRaWAN scalability. The ADR mechanism adapts the data rate setting based on the received signal strength index (RSSI) to increase the data rate while preserving the coverage. We can utilize the relay to divide a single slow data rate transmission into multiple fast data rate transmission to increase network scalability further. This approach will use the network resources more efficiently as decreasing a step of SF cuts the transmission time doubled [35].

In this evaluation, we use network topology 2 to compare the performance of network reliability and scalability. Not only comparing our proposed solution with fixed-path based forwarding, we evaluate the relay performance when exploiting the benefit of packet duplication to increase the network reliability. The mechanism took a small part of the cooperative relaying scheme [7] without calculating redundancy allocation; we named it CPR. While OOD, OODC, and FP include the relay for ADR selection, CPR only considers gateway as the destination. In CPR, the relay selects and combines packets randomly before transmission. Following 1% duty cycle on a relay running on SF 9 and BW 125 kHz, the relay has to transmit at max ten packets (20B for each packet) for approximately 1 s. Then, it has to wait for 100 s for the next transmission.

**Figure 9:**
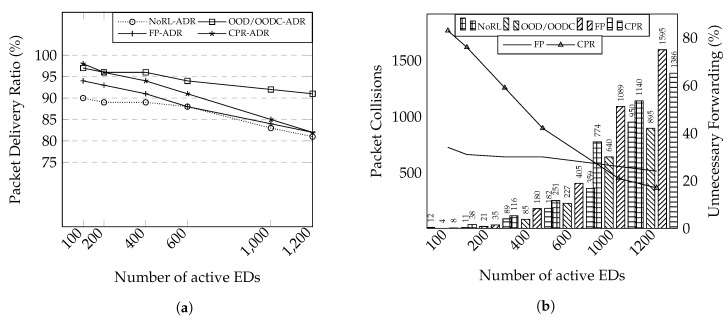
Performance evaluations on network topology 2. (**a**) PDR results. (**b**) The number of packet collision and unnecessary forwarding.

The results is shown in Figure 9a. As expected, ADR greatly improves performance compared to the previous evaluation. In this result, we are using 95% confidence interval, which resulted in less than 1 margin of error. Our mechanism shows reliability improvement above 90% of PDR. Conversely, both FP and CPR reliability performance degrade faster, although they provide good reliability for a small number of end-devices. In this evaluation, the difference in packet delivery ratio for the ADR mechanism is not that great. This is mainly caused by multiple channel utilization. The spreading factor for each end-device is different according to the distance. As the spreading factor in LoRa is orthogonal, the packet collision between relay and end-device highly occurs when they use a similar spreading factor.

#### 6.3.4. Unnecessary Forwarding Issue

We investigated the performance degradation in the previous experiment. The left-axis in Figure 9b shows the number of packet collisions in the gateway, while the right-axis tells the percentage of unnecessary forwarding resulted by FP and CPR. The results reveal that our work can minimize the number of packet collisions by preventing packet duplication.

Without a relay, the faraway end-devices have to use slow data rate settings to reach the gateway. Consequently, the transmission takes more time, blocking other end-devices to use the time resource. However, the FP forwarding shows a high amount of packet collision, although it can use a relay to utilize multiple faster data rates. This packet collision is probably caused by unnecessary forwarding. As per the approximate results, 30% of transmissions correspond to unnecessary forwarding. The amount of unnecessary forwarding is decreasing owing to the packet loss. Similarly, CPR achieves high reliability by packet redundancy. However, as the number of end-devices increases, the benefit of packet redundancy is not very effective. If we compare the reliability results in Figure 9a and the number of packet collisions, there is a trade-off between network reliability and efficiency. Although packet duplication by a forwarding mechanism can increase the network reliability, the unnecessary transmission can degrade the network capacity.

#### 6.3.5. Energy Consumption Issue

**Figure 10:**
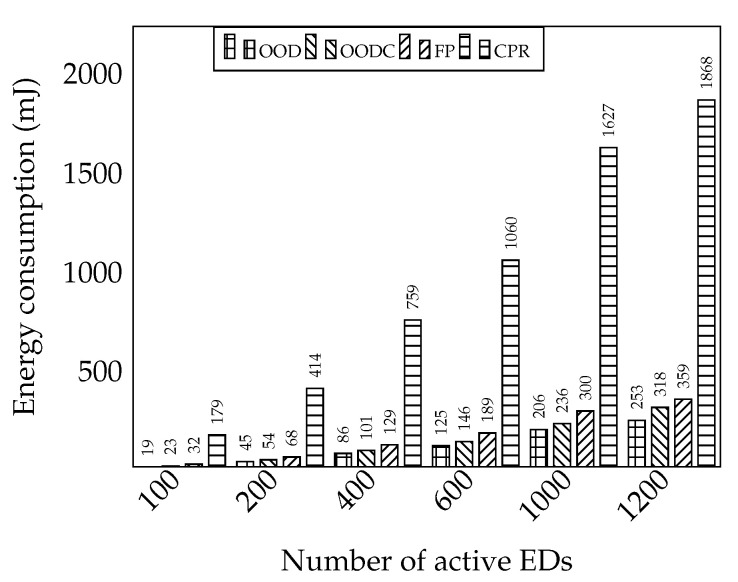
Measurement of energy consumption.

Based on the previous experiment, we compare the energy consumption for each approach, as shown in Figure 10. This energy measurement is based on the time on-air (ToA) of the forwarded packet. Hence, the distance and the packet size will affect energy consumption. For calculating energy, we use transmission power 90 mA and voltage 3 V.

(7) $$E=\sum_{n=1}^{k}ToA*mA*V$$

As shown in the figure, our OOD and OODC consume less energy for transmission. Compared to FP, both OOD and OODC forward packet efficiently so that it achieves good reliability. The OODC energy consumption is slightly higher than the OOD because of the header overhead and more transmissions needed. For CPR, it incurs high energy consumption as it combines several packets, makes the relay packet get bigger, and, consequently, the packet needs to be transmitted longer.

#### 6.3.6. OOD vs. OODC

The previous experiment demonstrates that both OOD and OODC approaches have similar reliability performance owing to the packet loss recovery strategy. The OOD mechanism allows the relay to retransmit a packet that is missing in the feedback message. Conversely, the OODC mechanism recovers the packet loss by applying network coding. Although their reliability is similar, the network coding preserves the reliability efficiently. We conducted an experiment based on topology 1 with the ADR mechanism to investigate it.

**Figure 11:**
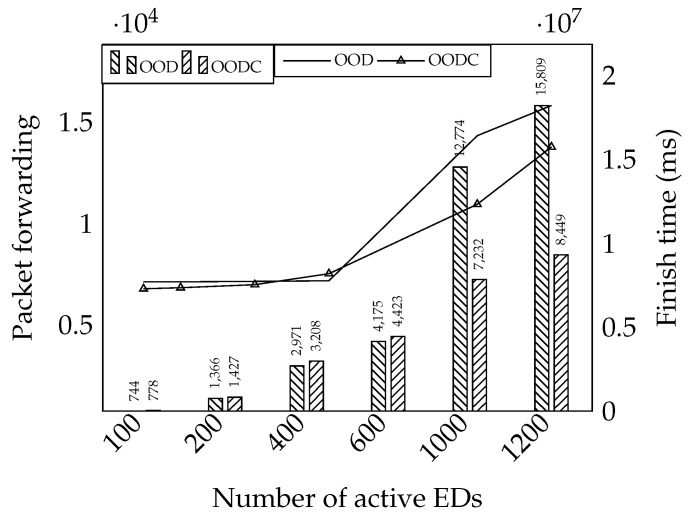
Performance evaluations between OD and OODC.

As shown in Figure 11, the left-axis is the amount of packet forwarding, and the right-axis is the finish time. Basically, the OODC mechanism forwards an additional encoded packet to guarantee successful decoding. However, the OODC forwards the packet effectively as it can recover the encoded packet during the feedback period. Conversely, the OOD failed to work properly when the end-devices increase. The bottleneck in the relay makes it challenging to finish the current transmission before the next feedback arrives. Those packets are transmitted again in the next feedback because they are late to be included in the feedback message. From this evaluation, we can conclude that network coding makes our mechanism increase reliability while preserving the network capacity.

## 7. Conclusions and Future Works

We presented opportunistic and on-demand (OOD) and opportunistic and on-demand network coding (OODC) forwarding to extend LPWAN coverage and increase its reliability while not degrading the network capacity. From the conducted experiment, we found out that the network resources are essential in LPWAN. Therefore, it is necessary to optimize the use of the network resources efficiently for better network capacity. Our method successfully reduces unnecessary forwarding, a negative effect of typical forwarding, primarily observed in long-range transmission. Combined with the network coding approach with a header overhead compression, it effectively achieves good reliability performance. Consequently, both OOD and OODC consume less energy consumption.

A forwarding mechanism that overcomes the hidden node problem should be considered in future work, particularly for transmission between relays. Moreover, RSSI-based priority is insufficient for facilitating improved reliability and efficiency. Without considering the workload, it can put a burden on a particular relay. The assumption of unlimited buffer size is also an issue in the current work. Lastly, a physical test for proof-of-concept validation is required.

## Figures and Tables

**Table 1:** Long-range radio (LoRa)WAN configurations for different data-rate scenarios.

Parameter	SDR	FDR	ADR
Transmission Power (dBm)	14	14	14
Carrier Frequency (MHz)	868	868	868
Bandwidth (kHz)	125	500	125
Spreading Factor	12	7	7–12
Coding Rate	4/5	4/5	4/5
Payload Size (byte)	20	20	20

## References

[1] Sanchez-Iborra R., Cano M.D. (2016). State of the art in LP-WAN solutions for industrial IoT services. Sensors.

[2] LoRa Alliance^®^. http://www.lora-alliance.org/.

[3] Sornin N., Luis M., Eirich T., Kramp T., Hersent O. (2015). Lorawan specification. LoRa Alliance.

[4] Augustin A., Yi J., Clausen T., Townsley W. (2016). A study of LoRa: Long range & low power networks for the internet of things. Sensors.

[5] Bor M.C., Roedig U., Voigt T., Alonso J.M. Do LoRa low-power wide-area networks scale?. Proceedings of the 19th ACM International Conference on Modeling, Analysis and Simulation of Wireless and Mobile Systems.

[6] Barrachina-Muñoz S., Adame T., Bel A., Bellalta B. Towards Energy Efficient LPWANs through Learning-based Multi-hop Routing. Proceedings of the 2019 IEEE 5th World Forum on Internet of Things (WF-IoT).

[7] Borkotoky S.S., Schilcher U., Bettstetter C. Cooperative Relaying in LoRa Sensor Networks. Proceedings of the 2019 IEEE Global Communications Conference (GLOBECOM).

[8] Biswas S., Morris R. ExOR: Opportunistic multi-hop routing for wireless networks. Proceedings of the 2005 Conference on Applications, Technologies, Architectures, and Protocols for Computer Communications.

[9] Adame Vázquez T., Barrachina-Muñoz S., Bellalta B., Bel A. (2018). HARE: Supporting efficient uplink multi-hop communications in self-organizing LPWANs. Sensors.

[10] Dias J., Grilo A. (2018). LoRaWAN multi-hop uplink extension. Procedia Comput. Sci..

[11] Sartori B., Thielemans S., Bezunartea M., Braeken A., Steenhaut K. Enabling RPL multihop communications based on LoRa. Proceedings of the 2017 IEEE 13th International Conference on Wireless and Mobile Computing, Networking and Communications (WiMob).

[12] Lundell D., Hedberg A., Nyberg C., Fitzgerald E. A Routing Protocol for LoRA Mesh Networks. Proceedings of the 2018 IEEE 19th International Symposium on “A World of Wireless, Mobile and Multimedia Networks” (WoWMoM).

[13] Bezunartea M., Van Glabbeek R., Braeken A., Tiberghien J., Steenhaut K. Towards Energy Efficient LoRa Multihop Networks. Proceedings of the 2019 IEEE International Symposium on Local and Metropolitan Area Networks (LANMAN).

[14] Bor M., Vidler J., Roedig U. LoRa for the Internet of Things. Proceedings of the International Conference on Embedded Wireless Systems and Networks (EWSN).

[15] Liao C., Zhu G., Kuwabara D., Suzuki M., Morikawa H. (2017). Multi-Hop LoRa Networks Enabled by Concurrent Transmission. IEEE Access.

[16] Sharma A., Kumar R. Performance comparison and detailed study of AODV, DSDV, DSR, TORA and OLSR routing protocols in ad hoc networks. Proceedings of the 2016 Fourth International Conference on Parallel, Distributed and Grid Computing (PDGC).

[17] Parvathi P. Comparative analysis of CBRP, AODV, DSDV routing protocols in mobile Ad-hoc networks. Proceedings of the 2012 International Conference on Computing, Communication and Applications.

[18] Broch J., Maltz D.A., Johnson D.B., Hu Y.C., Jetcheva J. A performance comparison of multi-hop wireless ad hoc network routing protocols. Proceedings of the 4th annual ACM/IEEE international conference on Mobile computing and networking.

[19] Ahmeda S.S., Esseid E.A. Review of routing metrics and protocols for wireless mesh network. Proceedings of the 2010 Second Pacific-Asia Conference on Circuits, Communications and System.

[20] Li Y., Liu Y., Luo P. Link Probability Based Opportunistic Routing Metric in Wireless Network. Proceedings of the 2009 WRI International Conference on Communications and Mobile Computing.

[21] Trakadas P., Zahariadis T., Voliotis S., Karkazis P., Velivassaki T.H., Sarakis L. Routing metric selection and design for multi-purpose WSN. Proceedings of the 21st International Conference on Systems, Signals and Image Processing (IWSSIP 2014).

[22] Rajkumar G., Kasiram R., Parthiban D. Optimized QoS metrics and performance comparison of DSR and AODV routing protocols. Proceedings of the IEEE-International Conference on Advances in Engineering, Science and Management (ICAESM -2012).

[23] Ferrari F., Zimmerling M., Thiele L., Saukh O. Efficient network flooding and time synchronization with Glossy. Proceedings of the 10th ACM/IEEE International Conference on Information Processing in Sensor Networks.

[24] Tseng Y.C., Ni S.Y., Chen Y.S., Sheu J.P. (2002). The broadcast storm problem in a mobile ad hoc network. Wirel. Netw..

[25] Wang F., Li D., Zhao Y. (2011). Analysis of csma/ca in IEEE 802.15.4. IET Commun..

[26] Rozner E., Seshadri J., Mehta Y., Qiu L. (2009). SOAR: Simple opportunistic adaptive routing protocol for wireless mesh networks. IEEE Trans. Mob. Comput..

[27] Katti S., Rahul H., Hu W., Katabi D., Médard M., Crowcroft J. (2008). XORs in the air: Practical wireless network coding. IEEE/ACM Trans. Netw..

[28] Mai D.L., Kim M.K. (2020). Multi-Hop LoRa Network Protocol with Minimized Latency. Energies.

[29] Tanjung D., Byeon S., Kim D.H., Deok Kim J. OODC: An Opportunistic and On-Demand Forwarding Mechanism for LPWA Networks. Proceedings of the 2020 International Conference on Information Networking (ICOIN).

[30] Gligoroski D., Kralevska K., verby H. Minimal header overhead for random linear network coding. Proceedings of the 2015 IEEE International Conference on Communication Workshop (ICCW).

[31] Li S., Ramamoorthy A. (2010). Improved compression of network coding vectors using erasure decoding and list decoding. IEEE Commun. Lett..

[32] LoRaWAN^®^ Regional Parameters v1.1rA: LoRa Alliance™. https://lora-alliance.org/resource-hub/lorawanr-regional-parameters-v11ra.

[33] Rappaport T.S. (1996). Wireless Communications: Principles and Practice.

[34] Petajajarvi J., Mikhaylov K., Roivainen A., Hanninen T., Pettissalo M. On the coverage of LPWANs: Range evaluation and channel attenuation model for LoRa technology. Proceedings of the 2015 14th International Conference on ITS Telecommunications (ITST).

[35] Semtech LoRa™ Modulation Basics, AN1200. 22. https://www.semtech.com/products/wireless-rf/lora-transceivers.

[36] Westlake J.R. (1968). A Handbook of Numerical Matrix Inversion and Solution of Linear Equations.

[37] Semtech SX1272 Dataset. https://www.semtech.com/products/wireless-rf/lora-transceivers/sx1272.

[38] Noreen U., Clavier L., Bounceur A. LoRa-like CSS-based PHY layer, Capture Effect and Serial Interference Cancellation. Proceedings of the 24th European Wireless Conference on European Wireless 2018.

